# Critical neutralizing fragment of Zika virus EDIII elicits cross-neutralization and protection against divergent Zika viruses

**DOI:** 10.1038/s41426-017-0007-8

**Published:** 2018-01-24

**Authors:** Wanbo Tai, Lei He, Yufei Wang, Shihun Sun, Guangyu Zhao, Chuming Luo, Pei Li, Haiyan Zhao, Daved H. Fremont, Fang Li, Shibo Jiang, Yusen Zhou, Lanying Du

**Affiliations:** 1 Lindsley F. Kimball Research Institute, New York Blood Center, New York, NY 10065 USA; 2 State Key Laboratory of Pathogen and Biosecurity, Beijing Institute of Microbiology and Epidemiology, 100071 Beijing, China; 3 Graduate School of Guangxi Medical University, 530021 Nanning, Guangxi China; 4 Department of Veterinary and Biomedical Sciences, University of Minnesota, Saint Paul, MN 55108 USA; 5 Department of Pathology and Immunology, Washington University School of Medicine, Saint Louis, MO 63110 USA; 6 Key Laboratory of Medical Molecular Virology of Ministries of Education and Health, Shanghai Medical College and Institute of Medical Microbiology, Fudan University, 200032 Shanghai, China

## Abstract

Zika virus (ZIKV) infection remains a serious health threat due to its close association with congenital Zika syndrome (CZS), which includes microcephaly and other severe birth defects. As no vaccines are available for human use, continuous effort is needed to develop effective and safe vaccines to prevent ZIKV infection. In this study, we constructed three recombinant proteins comprising, respectively, residues 296–406 (E296-406), 298–409 (E298-409), and 301–404 (E301-404) of ZIKV envelope (E) protein domain III (EDIII) fused with a C-terminal Fc of human IgG. Our results demonstrated that E298-409 induced the highest titer of neutralizing antibodies against infection with nine ZIKV strains isolated from different hosts, countries, and time periods, and it maintained long-term anti-ZIKV immunogenicity to induce neutralizing antibodies. Pups born to mice immunized with E298-409 were fully protected against lethal challenge with two epidemic human ZIKV strains, 2015/Honduras (R103451) and 2015/Colombia (FLR). Passive transfer of anti-E298-409 mouse sera protected pups born to naive mice, as well as type I interferon receptor-deficient adult A129 mice, from lethal challenge with human ZIKV strains R103451 and FLR, and this protection was positively correlated with neutralizing antibodies. These data suggest that the critical neutralizing fragment (i.e., a fragment that can induce highly potent neutralizing antibodies against divergent ZIKV strains) of ZIKV EDIII is a good candidate for development as an effective and safe ZIKV subunit vaccine to protect pregnant mothers and their fetuses against ZIKV infection. The E298-409-specific antibodies can be used for passive immunization to prevent ZIKV infection in newborns or immunocompromised adults.

## Introduction

Zika virus (ZIKV) is an emerging viral pathogen associated with severe neurological diseases, including Guillain–Barre Syndrome (GBS)^1,2^ and congenital Zika syndrome (CZS), which includes microcephaly, brain abnormalities, and other severe birth defects^3–7^. Increasing numbers of women have been infected with ZIKV during pregnancy and have given birth to newborns with congenital defects^7–10^, leading to serious consequences. A variety of ZIKV vaccines, such as those based on inactivated or live-attenuated viruses, viral vectors, DNA, RNA, and viral proteins^11–17^, have been developed against ZIKV infection in experimental animal models, and several have progressed to clinical trials^18–20^. To date, however, no vaccines are available for human use, a situation calling for a continuous effort to develop effective and safe vaccines against ZIKV infection.

ZIKV belongs to the same *Flaviviridae* family as dengue virus (DENV), West Nile virus (WNV), Japanese encephalitis virus (JEV), yellow fever virus (YFV), and tick-borne encephalitic virus (TBEV)^21,22^. The full-length genome of ZIKV encodes structural proteins, such as capsid (C), membrane (M) or precursor membrane (prM), and envelope (E), in addition to non-structural proteins (NS1, NS2a, NS2b, NS3, NS4a, NS4b, and NS5)^21,22^, among which E protein is the major protein for inducing neutralizing antibodies against ZIKV infection^23,24^ and, thus, a main target for developing ZIKV vaccines. ZIKV E protein consists of domain I (DI), domain II (DII), domain III (DIII), a stalk region, and a transmembrane domain (Supplementary Figure S1A)^25^. A number of ZIKV E-based vaccines were developed targeting prM-E or full-length E^11,14,16,26^, whereas a few have attempted to target fragments of ZIKV E protein domains, particularly EDIII, to identify important regions in the E protein for developing more effective and safer ZIKV vaccines^27^.

Although ZIKV E protein is a key target for developing ZIKV vaccines, studies have found that antibodies targeting ZIKV EDI/II are generally cross-reactive and poorly neutralizing, potently enhancing ZIKV and DENV infection, whereas those targeting ZIKV EDIII have the most potent neutralizing activity against ZIKV infection^28^. In addition, antibodies targeting DENV and/or WNV E proteins, including the fusion loop region, may bind and cross-react with ZIKV EDI/II region, but they do not neutralize ZIKV infectivity, thus promoting antibody-dependent enhancement (ADE)^29–31^.

In this study, we constructed three recombinant proteins comprising ZIKV EDIII fragments of different lengths and found that one of the ZIKV EDIII fragments, E298-409, could induce potent neutralizing antibodies, which protected newborn mice and type I interferon receptor-deficient adult A129 mice from lethal challenge with divergent ZIKV strains. The present study demonstrates that the identified EDIII fragment can be further developed as an effective and safe vaccine for prevention of ZIKV infection and that the anti-E298-409 antibodies can be used for pre-exposure prophylaxis of ZIKV infection.

## Materials and methods

### Ethics statement

Seven-day-old male and female BALB/c pups, 6- to 8-week-old female BALB/c mice, and 5-week-old male and female A129 mice were used in the study. The animal studies were carried out in strict accordance with the recommendations in the Guide for the Care and Use of Laboratory Animals of the National Institutes of Health. The protocols were approved by the Committee on the Ethics of Animal Experiments of the New York Blood Center (Permit Numbers: 344.00 and 345.00).

### Construction, expression, and purification of recombinant proteins

Recombinant ZIKV EDIII fragments were prepared as previously described with some modifications^32,33^. Briefly, the genes encoding the respective ZIKV EDIII fragments were amplified by PCR using a codon-optimized ZIKV E (ZikaSPH2015 strain, GenBank accession no. KU321639.1) plasmid as the template, followed by fusion with a C-terminal Fc of human IgG (hereinafter E296-406, E298-409, or E301-404). Recombinant ZIKV E (residues 1–454) fused with a C-terminal Fc (E1-454) was constructed as described above. Recombinant ZIKV EDIII (residues 298–409) containing a C-terminal foldon sequence and a His_6_ tag (EDIII-His) was constructed using the pJW4303 expression vector^34^. The aforementioned plasmids were transfected into 293T cells, and the respective proteins were harvested from the cell culture supernatants and purified by Protein A affinity chromatography (for the Fc-tagged proteins) (GE Healthcare, Port Washington, NY, USA) or Ni-NTA Superflow (for the His-tagged protein) (Qiagen, Germantown, MD, USA).

### SDS-PAGE and western blot

The purified EDIII fragments were analyzed by SDS-PAGE and Western blot as previously described^32,35^. Briefly, the proteins (boiled or non-boiled) were separated by 10% Tris-Glycine SDS-PAGE gels and stained by Coomassie Brilliant Blue or transferred to nitrocellulose membranes for Western blot analysis. The blots were blocked with 5% fat-free milk in PBST overnight at 4 °C and incubated for 1 h at room temperature with a ZIKV EDIII-specific mouse monoclonal antibody (mAb), ZV-54 (0.4 μg/ml)^36^. After three washes, the blots were incubated with horseradish peroxidase (HRP)-conjugated goat anti-mouse IgG (1:5000, Invitrogen, Waltham, MA, USA) for 1 h at room temperature. The signals were detected with ECL Western blot substrate buffer and Amersham Hyperfilm (GE Healthcare).

### Animal immunization

This procedure was carried out as previously described^32,35^. Briefly, female BALB/c mice were intramuscularly (i.m.) immunized with EDIII protein fragments (10 μg per mouse) or PBS control, in the presence of aluminum (Alum, 500 μg per mouse) and monophosphoryl lipid A (MPLA, 100 μg per mouse) adjuvants (InvivoGen, San Diego, CA, USA), and boosted with the same immunogens at day 21, day 42, month 7, and month 10, respectively. Sera were collected at months 0, 2, 7, and 10 post immunization to test antibodies and neutralizing antibodies.

### ELISA

Mouse sera were analyzed for ZIKV EDIII-specific antibody responses by ELISA as previously described^32,35^. Briefly, ELISA plates were coated with ZIKV EDIII-His or E1-454 protein overnight at 4 °C and blocked with 2% fat-free milk for 2 h at 37 °C. Serially diluted mouse sera were added to the plates and then incubated for 1 h at 37 °C. After washing, the plates were incubated with HRP-conjugated anti-mouse IgG (1:5000), anti-mouse IgG1 (1:2000, Bethyl Laboratories, Montgomery, TX, USA), or anti-mouse IgG2a (1:2000, Invitrogen) for 1 h at 37 °C. The reaction was detected by 3,3′,5,5′-tetramethylbenzidine (TMB) substrate (Invitrogen) and stopped by 1 N H_2_SO_4_. The absorbance at 450 nm was measured using an ELISA plate reader (Tecan, Morrisville, NC, USA), and the respective antibody titers were calculated.

### ZIKV preparation and plaque reduction neutralization test (PRNT)

ZIKV (human strains, including FLR (2015/Colombia), R103451 (2015/Honduras), PAN2015 (2015/Panama), PAN2016 (2016/Panama), PRVABC59 (2015/Puerto Rico), PLCal_ZV (2013/Thailand), IbH 30656 (1968/Nigeria), mosquito strain MEX 2-81 (2016/Mexico), and rhesus strain MR 766 (1947/Uganda)) was amplified in Vero E6 cells, and the related viral titers were determined by a standard plaque-forming assay. A PRNT was then carried out to measure the titers of neutralizing antibodies in the collected mouse sera. Briefly, viral solutions of each ZIKV at ~100 plaque-forming units (PFU) were incubated with serially diluted sera (e.g., polyclonal antibody, pAb) or ZV-54 neutralizing mAb control^36^ for 1.5 h at 37 °C. The antibody-virus mixtures were transferred onto Vero E6 cells and incubated for 1 h at 37 °C. The cells were then overlaid with medium (1% carboxymethyl cellulose in DMEM containing 2% FBS), cultured at 37 °C for 4–5 days, and further stained with 0.5% crystal violet. The titers of neutralizing antibodies in mouse sera are presented as the highest dilution of sera resulting in a complete inhibition of virus infectivity in at least 50% of the wells (PRNT_50_). Each serum was tested in duplicate wells in fourfold serial dilutions from 1:10 to 1:2,560. The neutralizing activity of mAb was calculated in duplicate wells at 5-fold serial dilutions from 50 μg/ml and is expressed as 50% neutralization dose (ND_50_).

### ZIKV challenge in pups born to vaccinated female BALB/c mice

Seven-day-old pups born to female BALB/c mice vaccinated with ZIKV E296-406, E298-409, E301-404, and PBS, respectively, were challenged intraperitoneally (i.p.) with 5 × 10^4^ PFU of ZIKV R103451 or FLR strains in 50 μl solution. Mouse survival and weight were observed for 14 days post-viral infection (p.i.).

### Passive protection against ZIKV challenge in pups born to naive female BALB/c mice

Mouse sera immunized with the aforementioned ZIKV fragments were i.p. injected into 7-day-old naïve BALB/c pups (100 μl per pup). The sera were diluted in PBS to produce the same log ELISA titer of 4.00 (10^4^) after detection of the IgG titers using ZIKV EDIII-His protein. Six hours later, pups were i.p. challenged with 5 × 10^4^ PFU of ZIKV R103451 or the FLR strain as described above. Pups injected with ZIKV only were included as sham controls. Some pups also received another dose of sera 12 h post-challenge with virus. Mouse survival and weight were recorded for 14 days p.i.

### Passive protection against ZIKV challenge in lethal A129 mice

Five-week-old A129 mice were i.p. injected with sera (200 μl per mouse) (with a ZIKV EDIII-specific IgG titer of 10^5^ as described above) from E298-409-immunized mice. Six hours later, mice were i.p. challenged with 10^2^ or 10^3^ PFU of ZIKV R103451 or FLR strain. Mice challenged with 10^3^ PFU of ZIKV were further injected with the above sera (200 μl per mouse) at 12 h, or 12, 24, and 48 h p.i., respectively. Mice injected with ZIKV only were included as sham controls. Mouse survival and weight were evaluated for 14 days p.i. ZIKV RNA titers were detected from sera and tissues collected at 3 days p.i. as described below, and pathological changes were detected from tissues collected at 5 days p.i. by H&E staining as previously described^35,37^.

### Quantitative reverse transcriptase PCR (qRT-PCR)

ZIKV RNA copies in the sera and tissues of challenged mice were detected by qRT-PCR as previously described^38^. Briefly, ZIKV RNA was extracted using the QIAamp MinElute Virus Spin Kit (for sera) (Qiagen) and the RNeasy Mini Kit (for tissues) (Qiagen). The extracted ZIKV RNA was quantified using one-step qRT-PCR with Power SYBR Green PCR Master Mix, MultiScribe Reverse Transcriptase, and Ambion RNase Inhibitor (Thermo Fisher Scientific, Waltham, MA, USA) in the ViiA 7 Master Cycler PCR System (Thermo Fisher Scientific) according to the manufacturers’ instructions. The forward and reverse primers used for the amplification were 5′-TTGGTCATGATACTGCTGATTGC-3′ and 5′-CCTTCCACAAAGTCCCTATTGC-3′, respectively.

### Statistical analysis

The values are presented as the means with standard error (SE). Statistical significance among the different groups was calculated by Student’s two-tailed *t*-test using GraphPad Prism Statistical Software. *, **, and *** indicate *P* < 0.05, *P* < 0.01, and *P* < 0.001, respectively.

## Results

### ZIKV EDIII fragments, especially E298-409, induced broad-spectrum neutralizing antibodies and maintained long-term immunogenicity

Three ZIKV EDIII fragments comprising residues 296–406, 298–409, and 301–404, respectively, were constructed, and each of them was fused with a C-terminal Fc (Supplementary Figure S1B). Except for E301-404, which covers the entire sequence of EDIII (residues 301–404), both E296-406 and E298-409 have a few residues from the EDI and stalk regions (Supplementary Figure S1C). The recombinant proteins were expressed in 293T cell culture supernatants and purified with high purity. They formed dimeric structures and maintained good conformation (Supplementary Figure S2A). Particularly, these EDIII fragments were recognized by a mAb (ZV-54) specific to ZIKV EDIII protein (Supplementary Figure S2B)^36^, demonstrating their strong specificity to ZIKV.

**Fig. 1:**
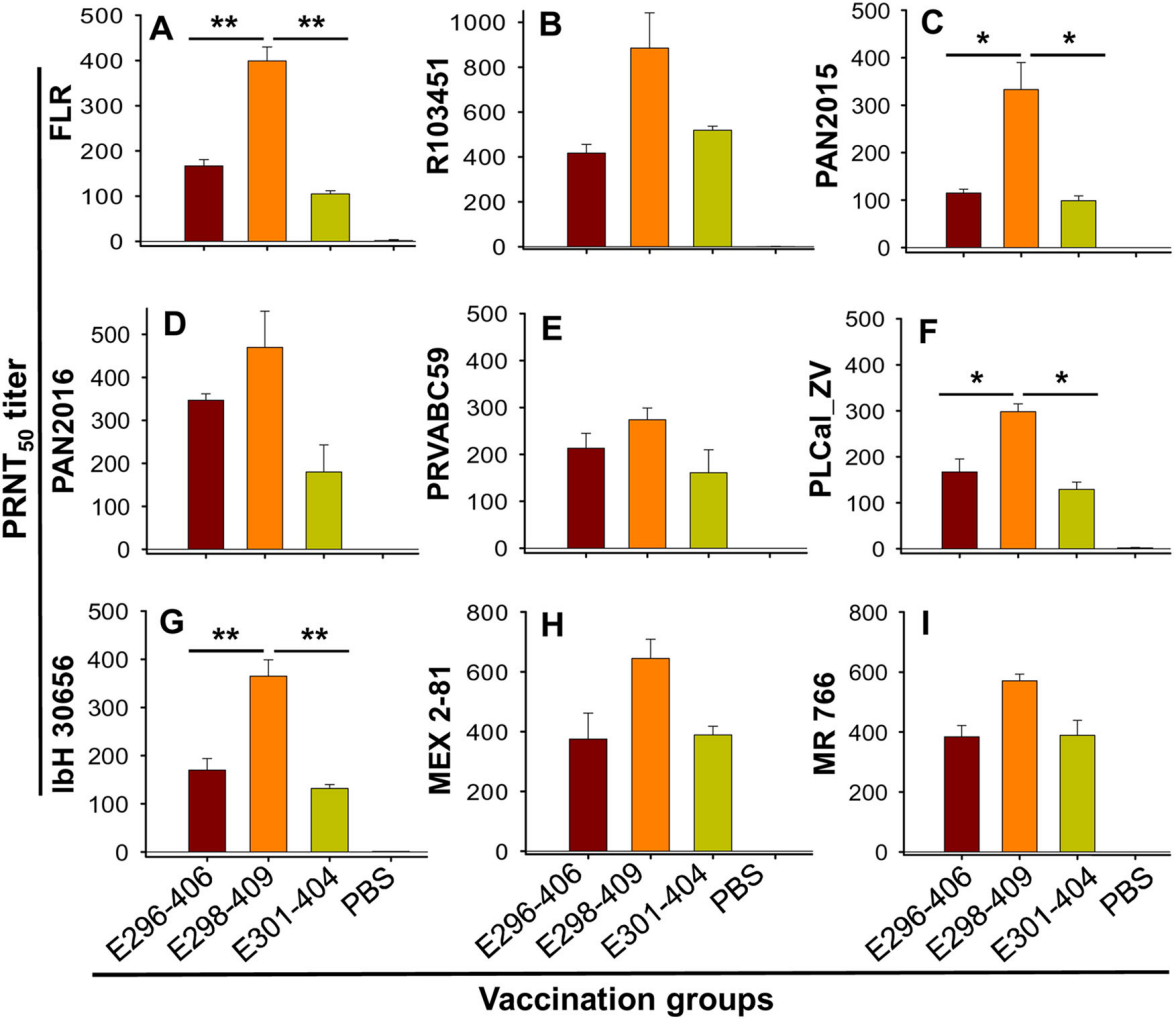
ZIKV EDIII fragments induced neutralizing antibodies against divergent human, mosquito, and rhesus strains of ZIKV Mouse sera from 2 months post immunization with ZIKV EDIII fragments or PBS control were tested by PRNT assay against infection with ZIKV strains isolated from human, including FLR (**a**), R103451 (**b**), PAN2015 (**c**), PAN2016 (**d**), PRVABC59 (**e**), PLCal_ZV (**f**), and IbH 30656 (**g**), as well as from mosquito, MEX 2-81 (**h**), and rhesus, MR 766 (**i**). Neutralizing activity is expressed as 50% neutralizing antibody titer (PRNT_50_), and the data are presented as the means ± SE of the mice in each group (*n* = 5). Significant differences were noted between E208-409 and the other two EDIII fragments against FLR (**P < 0.01), R103451 (*P < 0.05), PLCal_ZV (*P < 0.05), and IbH 30656 (**P < 0.01), respectively. The experiments were repeated twice, and similar results were obtained

To evaluate the immunogenicity of the three EDIII fragments, we used purified proteins to immunize female BALB/c mice following the immunization schedule described in Supplementary Figure S3A. Sera collected at 2 months after immunization were tested for cross-neutralization against nine ZIKV strains isolated from different hosts (human, mosquito, and rhesus), various countries and regions (Colombia, Honduras, Panama, Puerto Rico, Thailand, Nigeria, Mexico, and Uganda), and different periods of time (1947, 1968, 2013, 2015, and 2016) (Supplementary Figure S4A). These ZIKV strains belong to diverse phylogenetic groups (Supplementary Figure S4B) and have a number of amino acid variations among their E proteins (Supplementary Figure S4C). Compared with the E296-406 and E301-404 fragments, the E298-409 fragment induced higher titers of neutralizing antibodies able to cross-neutralize all ZIKV strains tested (Fig. 1), including those with different EDIII sequences, e.g., PRVABC59 (isolated from a patient in an American country), IbH 30656 (isolated from a patient in an African country), and MR 766 (isolated from a rhesus macaque in an African country), and those with the same EDIII sequences, e.g., FLR, R103451, PAN2015, PAN2016, MEX 2-81 (isolated from patients or mosquitoes in American countries), and PLCal_ZV (isolated from a patient in an Asian country) (Supplementary Figure S4). Specifically, the level of E298-409-elicited neutralizing antibodies was significantly higher than that induced by either E296-406 or E301-404 against epidemic human 2015 strains FLR (Fig. 1a) and PAN2015 (Fig. 1c), 2013 strain PLCal_ZV (Fig. 1f), and 1968 strain IbH 30656 (Fig. 1g). By comparison, no ZIKV-specific neutralizing antibody was detected in the control mice injected with PBS (Fig. 1). The above results demonstrated that ZIKV EDIII fragments, particularly E298-409, were capable of eliciting highly potent neutralizing antibodies against divergent human, mosquito, and rhesus ZIKV strains from different countries and years.

**Fig. 2:**
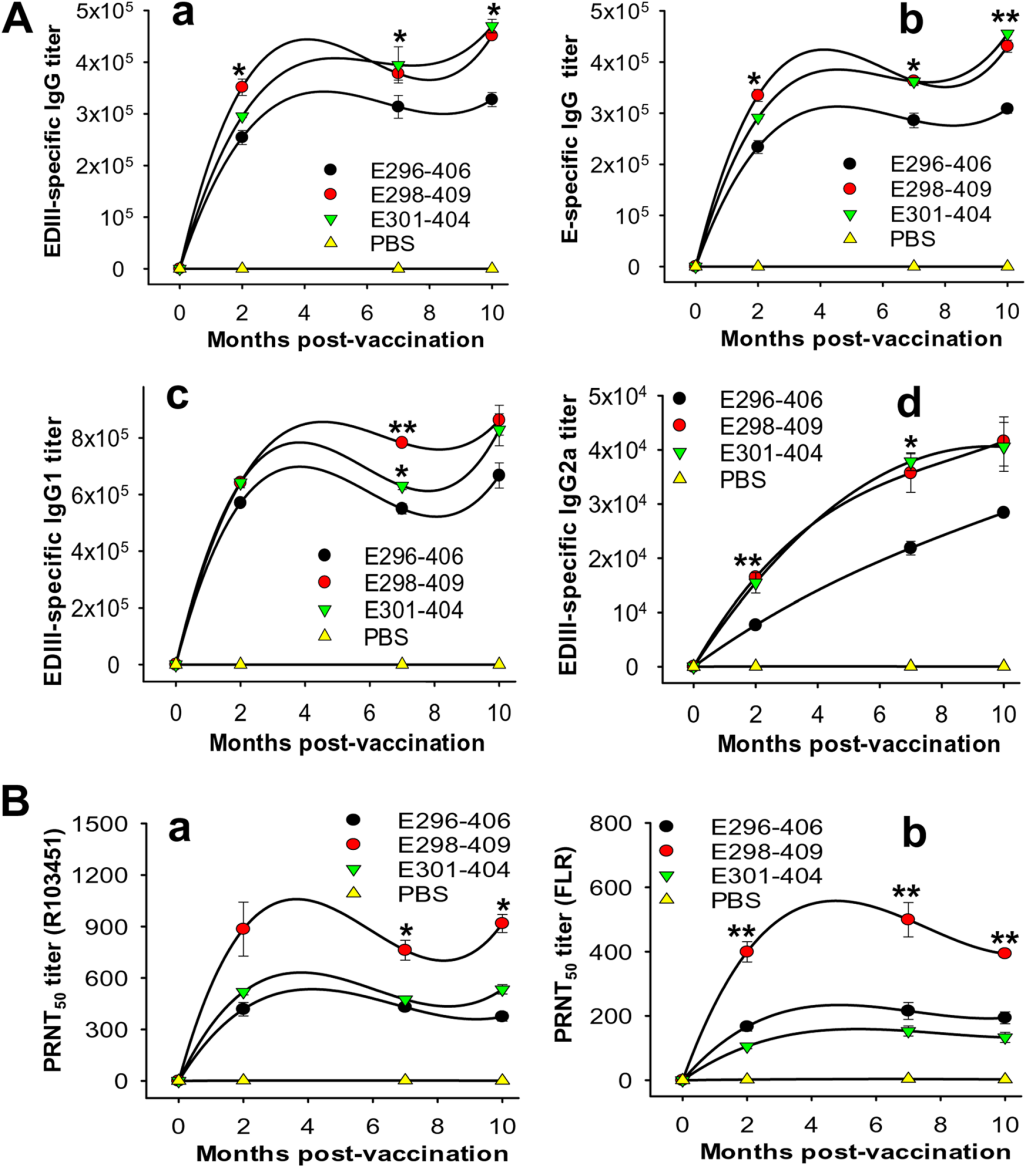
ZIKV EDIII fragments induced long-term antibody responses and neutralizing antibodies against infection with two epidemic human ZIKV strains (**A**) Mouse sera collected before immunization and 2, 7, and 10 months post immunization with ZIKV EDIII fragments or PBS control were tested by ELISA for ZIKV EDIII (a) and/or E (b)-specific IgG, IgG1 (c), and IgG2a (d) antibodies. The antibody titers were expressed as the endpoint dilutions that remained positively detectable and are presented as the means ± SE of the mice in each group (*n* = 5). For EDIII-specific IgG (a), significant differences were observed between E298-409 and E296-406 at 2, 7, and 10 months (*P < 0.05), and between E296-406 and E301-404 at 10 months (*P < 0.05) post immunization. For E-specific IgG (b), significant differences were found between E298-409 and E296-406 at 2, 7 (*P < 0.05), and 10 months (**P < 0.01), and between E296-406 and E301-404 at 7 (*P < 0.05) and 10 months (**P < 0.01) post immunization. For IgG1, significant differences were noted between E298-409 and E296-406 (**P < 0.01), and between E298-409 and E301-404 (*P < 0.05) at 7 months post immunization. For IgG2a, significant differences occurred between E298-408 and E296-406 at 2 months (**P < 0.01), and between E298-409 or E301-404 and E296-406 (*P < 0.05) at 7 months post immunization. (**B**) The aforementioned sera were tested by PRNT assay against infection with ZIKV R103451 (a) or FLR (b) strain. The neutralizing antibody titers were expressed as PRNT_50_ and are presented as the means ± SE of the mice in each group (*n* = 5). Significant differences occurred between E298-409 and the other two EDIII fragments against R103451 (*P < 0.05) and FLR (**P < 0.01), respectively, at the indicated months post immunization. The experiments were repeated twice, and similar results were obtained

To investigate the long-term immunogenicity and neutralizing activity of the ZIKV EDIII fragments, the immunized mice were observed for up to 10 months, and sera were collected at 0, 2, 7, and 10 months post immunization to test ZIKV EDIII and E-specific antibody responses and neutralizing antibodies against two selected epidemic human strains, R103451 and FLR. The data from the antibody test revealed that although all three EDIII fragments were able to elicit long-term EDIII-specific IgG antibodies, those induced by the E298-409 and E301-404 fragments showed significantly higher titers than that induced by the E296-406 fragment (Fig. 2Aa). These IgG antibodies were further tested using a ZIKV E protein containing residues 1–454, and the results showed that the E-specific IgG antibodies corresponded to the respective EDIII-specific IgG antibodies induced by the three EDIII fragments (Fig. 2Ab). Compared to E301-404, E298-409 elicited a relatively higher titer of EDIII-specific IgG1 (Th2-associated) antibodies (Fig. 2Ac) but a similar level of IgG2a (Th1-associated) antibodies (Fig. 2Ad). E296-406 induced the lowest titers of IgG1 and IgG2a antibodies among the three fragments in the test months (Fig. 2Ac, d). E298-409 elicited more potent long-term neutralizing antibody responses than E296-406 and E301-404 against ZIKV R103451 (Fig. 2Ba) and FLR (Fig. 2Bb) strains for at least 10 months (Fig. 2B). In contrast, control PBS induced only background levels of IgG, IgG1, and IgG2a antibody responses and neutralizing antibodies against the two tested ZIKV strains (Fig. 2). The above data indicate that ZIKV EDIII fragments, especially E298-409, maintained the immunogenicity required to elicit long-term EDIII and E-specific antibody responses, particularly neutralizing antibody responses against infection with variant epidemic human ZIKV strains.

### ZIKV EDIII fragments protected pups born to immunized BALB/c mice against challenge with divergent epidemic human ZIKV strains

Having confirmed the immunogenicity of ZIKV EDIII fragments, we next wanted to know whether the antibodies induced by these proteins could protect against ZIKV infection in animal models in vivo. Seven-day-old newborn mice (pups) are a convenient and effective means for evaluating the protective efficacy of ZIKV vaccines, as they are highly susceptible to ZIKV infection^17^. Therefore, we initially used pups born to immunized female BALB/c mice at 7 months post immunization for ZIKV challenge (Supplementary Figure S3B). Theoretically, if pups receive anti-ZIKV neutralizing antibodies from their mothers during pregnancy, they are expected to be protected against ZIKV infection. To test the protective efficacy of ZIKV EDIII fragments, pups were challenged with 5 × 10^4^ PFU of the aforementioned two epidemic human ZIKV strains, R103451 and FLR, and the resulting survival and weight, as well as the neutralizing antibodies from mothers and pups, were evaluated.

**Fig. 3:**
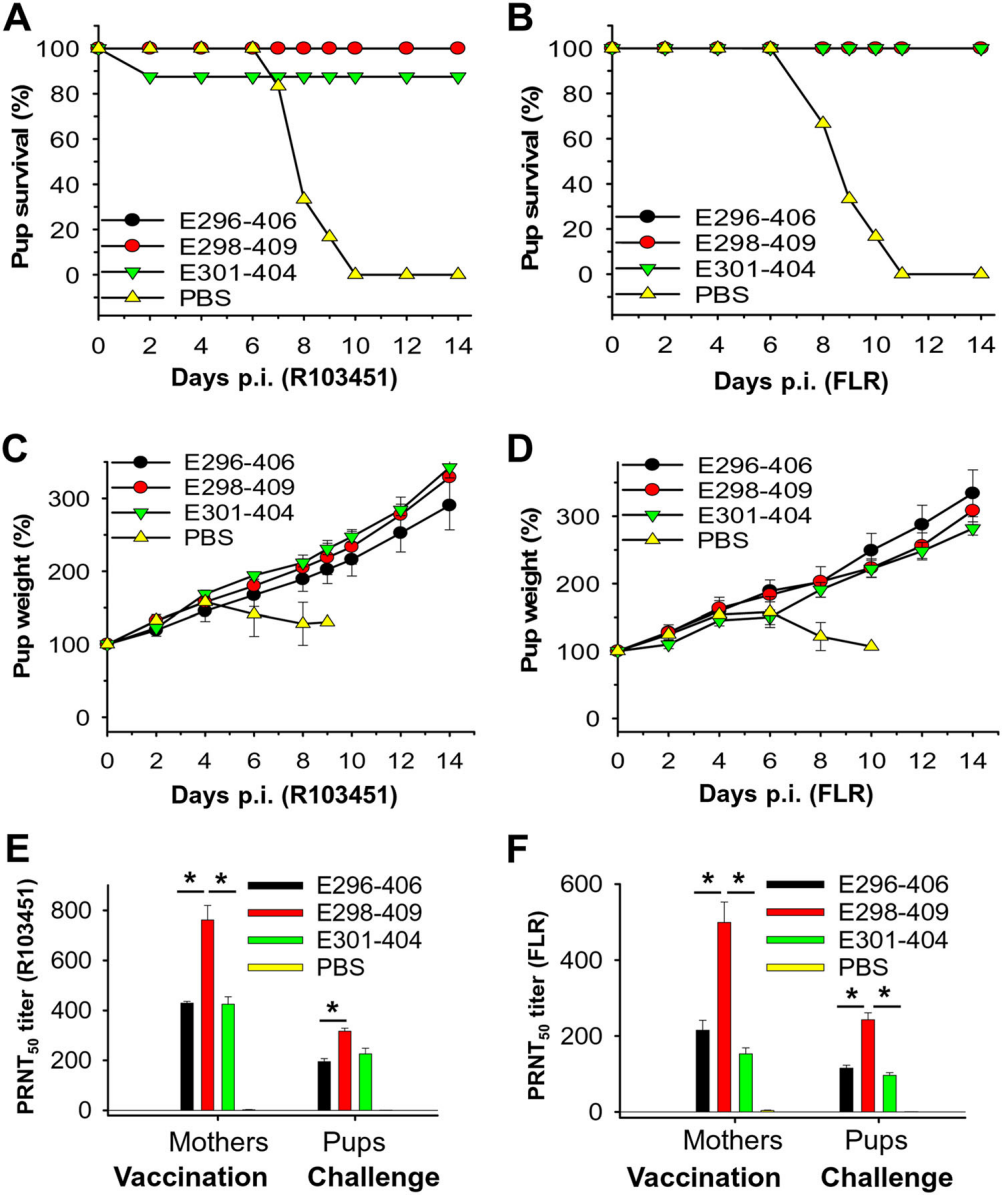
ZIKV EDIII fragments protected pups born to immunized BALB/c mice against challenge with two epidemic human ZIKV strains Groups of 7-day-old pups (*n* = 6) born to female BALB/c mice immunized with ZIKV EDIII fragments or PBS control were challenged with ZIKV (5 × 10^4^ PFU), and percentages of survival in the ZIKV R103451 (**a**) or FLR (**b**) strain treatments, as well as percentages of weight in the ZIKV R103451 (**c**) or FLR (**d**) treatments, were recorded for 14 days p.i. The data in **c** and **d** are presented as the means ± SE of surviving pups in each group. Neutralizing antibody titers of sera of mothers at the time of pregnancy and surviving pups at 14 days p.i. in the ZIKV R103451 (**e**) or FLR (**f**) strain treatments are expressed as PRNT_50_. The data are presented as the means ± SE of sera of pregnant mothers (*n* = 5) or surviving pups in each group, and the experiments were repeated twice with similar results. Significant differences (*P < 0.05) between E298-409 and E296-406 or E301-404 are shown

Except for the pups from mice immunized with E301-404, which had an ~83% survival rate against ZIKV R103451 challenge, all pups born to the E298-409- and E296-406-immunized mice survived the R103451 challenge (Fig. 3a), and all pups born to the E298-409-, E296-406-, and E301-404-immunized mice survived the FLR challenge (Fig. 3b). All pups born to the above three EDIII fragment-immunized mice had increased weights during the detection period (14 days) after challenge with both R103451 (Fig. 3c) and FLR (Fig. 3d) strains. The evaluation of the neutralizing antibodies revealed that, similar to the immunized mothers, pups from E298-409-immunized mice had the highest neutralizing antibody titers among the three treatments against R103451 (Fig. 3e) and FLR (Fig. 3f) strains. In contrast to the above results, pups born to the mice injected with PBS did not survive challenge with the R103451 (Fig. 3a) and FLR (Fig. 3b) strains, and all of them died at 10 and 11 days p.i., respectively, with decreased weights 4 and 6 days post-challenge with R103451 (Fig. 3c) or FLR (Fig. 3d). This result is mainly because no neutralizing antibodies were detected in the mothers injected with PBS (Fig. 3e, f). The above results demonstrated that pups born to mice immunized with ZIKV EDIII fragments, particularly E298-409, were protected against infection with divergent ZIKV strains, possibly because maternal neutralizing antibodies had been transferred via placenta from mothers to fetuses during pregnancy.

### Adoptive transfer of ZIKV E298-409-immunized mouse sera completely protected naive pups against challenge with divergent epidemic human ZIKV strains

**Fig. 4:**
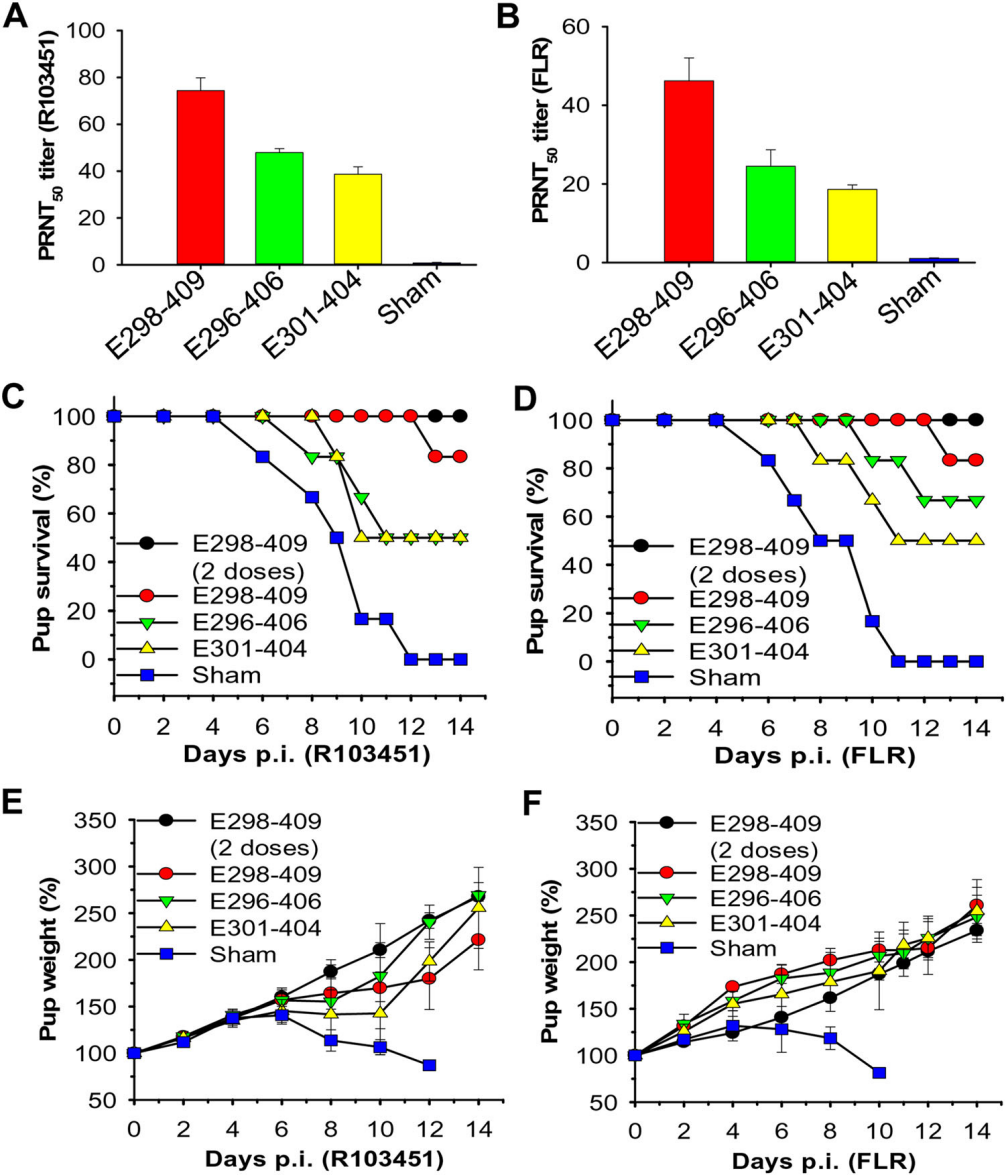
Adoptive transfer of ZIKV EDIII-immunized mouse sera protected pups born to naïve BALB/c mice against challenge with two epidemic human ZIKV strains Groups of 7-day-old pups (*n* = 6) born to naive BALB/c mice were adoptively transferred with sera of mice immunized with EDIII fragments and challenged with ZIKV (5 × 10^4^ PFU) 6 h later. The neutralizing antibody titers (PRNT_50_) from adoptively transferred sera in the ZIKV R103451 (**a**) or FLR (**b**) strain treatments are shown. All sera were adjusted to an equal titer (10^4^) of IgG antibody specific to ZIKV EDIII. Percentages of survival of pups challenged with ZIKV R103451 (**c**) or FLR (**d**), as well as percentages of weight of pups challenged with ZIKV R103451 (**e**) or FLR (**f**), were recorded for 14 days p.i. The data in **e** and **f** are presented as the means ± SE of surviving pups in each group. Pups infected with ZIKV only were included as sham controls. “Two doses” (in **c**–**f**) indicates that pups were adoptively transferred with two doses of the above sera 6 h before and 12 h after ZIKV infection

To elucidate the mechanism of ZIKV EDIII fragments in protecting against ZIKV infection and understand the correlation between neutralizing antibodies and protection, 7-day-old pups born to naive BALB/c mice were passively transferred with sera (equal to 10^4^ ZIKV EDIII-specific IgG titer) from mice immunized with ZIKV EDIII fragments. 6 h later, these pups were challenged with 5 × 10^4^ PFU of ZIKV R103451 or FLR, and then observed for survival and weight change. Pups transferred with anti-E298-409 sera, which had a higher neutralizing antibody titer than either the anti-E296-406 or anti-E301-404 sera against ZIKV R103451 (Fig. 4a) and FLR (Fig. 4b), exhibited the highest survival rates among the three groups after challenge with both ZIKV strains (Fig. 4c, d). As expected, pups receiving anti-E301-404 sera, which had the lowest neutralizing antibody titer against both R103451 and FLR (Fig. 4a, b), had the lowest survival rates among the three groups after challenge with R103451 and FLR (Fig. 4c, d). Clearly, pups receiving sera of mice immunized with all three EDIII fragments were able to maintain their body weight after challenge with both ZIKV strains (Fig. 4e, f). In contrast, pups in the sham controls did not survive ZIKV challenge, and all pups died at 12 and 11 days p.i., respectively, of R103451 and FLR strains (Fig. 4c, d), with continually decreasing weights after 6 days post-challenge with both ZIKV strains (Fig. 4e, f).

As one dose of anti-E298-409 sera could not fully protect the pups against ZIKV infection, pups were given an additional dose of these sera 12 h post-challenge with ZIKV. Interestingly, these pups were completely protected from R103451 and FLR challenge (Fig. 4c, d), and their weights steadily increased during the observation period (Fig. 4e, f). These data confirmed that the protection level in pups against ZIKV infection is associated with the amount of the neutralizing antibodies induced by the ZIKV EDIII fragments.

### Protective efficacy of E298-409 of ZIKV EDIII against divergent epidemic human ZIKV strains in ZIKV-susceptible A129 mice

**Fig. 5:**
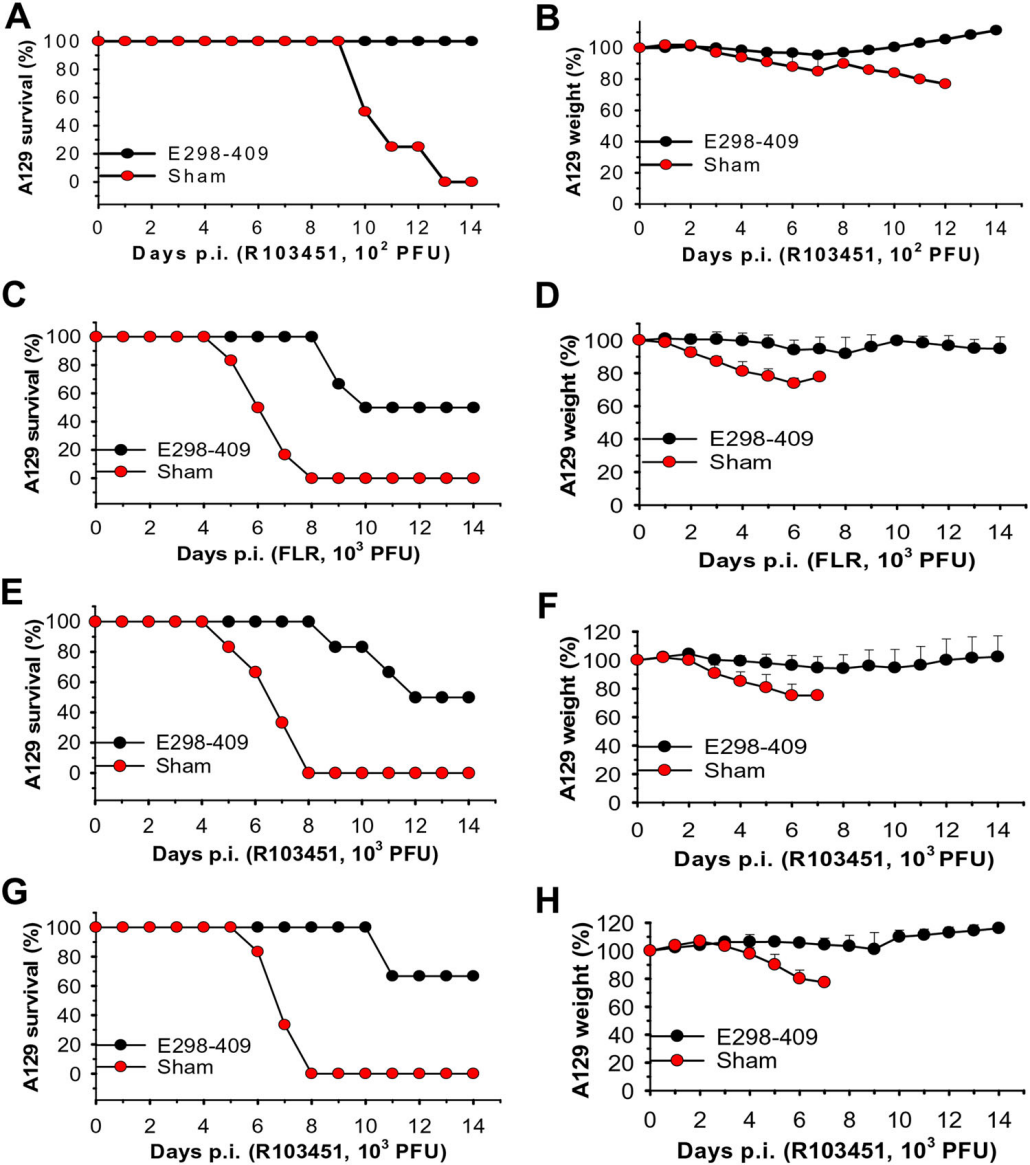
Adoptive transfer of sera of mice immunized with E298-409 of ZIKV EDIII protected lethal A129 mice against challenge with two epidemic human ZIKV strains The following three experiments were carried out in 5-week-old A129 mice (*n* = 6). Mice infected with ZIKV only were included as sham controls. (1) Mice were passively transferred with anti-E298-409 sera (e.g., 10^5^ ZIKV EDIII-specific IgG antibody titer), then challenged with ZIKV R103451 (10^2^ PFU) 6 h later, and percentages of survival (**a**) and weight (**b**) were calculated for 14 days p.i. (2) Mice were passively transferred with the above sera 6 h before and 12 h after infection with the ZIKV FLR (**c**,** d**) or R103451 (**e**,** f**) (10^3^ PFU) strains, and percentages of survival were recorded for FLR (**c**) and R103451 (**e**), as well as weights from FLR (**d**) and R103451 (**f**), for 14 days p.i. (3) Mice were passively transferred with the above sera 6 h before and 12, 24, and 48 h post-challenge with ZIKV R103451 (10^3^ PFU), and percentages of survival (**g**) and weight (**h**) were recorded for 14 days p.i. The data in **b**, **d**, **f**, and **g** are presented as the means + SE of surviving mice in each group

Given that the pups born to the mother mice immunized with E298-409 and the pups receiving anti-E298-409 sera were fully protected from ZIKV infection, we then tested whether anti-E298-409 sera could also protect type I interferon receptor-deficient adult A129 mice, which are highly susceptible to ZIKV infection, against lethal ZIKV challenge. Anti-E298-409 sera equal to 10^5^ ZIKV EDIII-specific IgG titer and neutralizing antibody titers of 3.6 × 10^2^ and 1.3 × 10^2^, respectively, against R103451 and FLR infection, were adoptively transferred into adult A129 mice, and survival and weight change were recorded in the challenged mice. Three different challenge experiments were performed in A129 mice. First, mice received sera 6 h before infection with 10^2^ PFU of ZIKV R103451 (Fig. 5a, b). Second, mice received sera 6 h before and 12 h after infection with a higher titer (10^3^ PFU) of ZIKV FLR (Fig. 5c, d) or R103451 (Fig. 5e, f). Third, mice received sera 6 h before and 12, 24, and 48 h after infection with 10^3^ PFU of ZIKV R103451 (Fig. 5g, h).

The results of the first experiment demonstrated that, while the sham control mice did not survive from ZIKV challenge (10^2^ PFU) and showed increased weight loss, A129 mice receiving anti-E298-409 neutralizing sera had 100% survival after ZIKV R103451 infection (Fig. 5a) with increased weight after challenge (Fig. 5b). This result suggests that anti-E298-409 antibodies can also protect immunocompromised adult mice from ZIKV infection. The data from the second experiment revealed that A129 mice passively transferred with anti-E298-409 sera were protected against challenge with a higher titer (10^3^ PFU) of ZIKV FLR (Fig. 5c, d) or R103451 (Fig. 5e, f) strains. The results of the third experiment indicated that increased doses of passively transferred anti-E298-409 sera with neutralizing activity enhanced A129 mouse survival (Fig. 5g) and slightly increased weight (Fig. 5h) after R103451 challenge. In both the second and third experiments, all control A129 mice receiving 10^3^ PFU of ZIKV R103451 or FLR died at 8 days p.i. (Fig. 5c, e, and g) with decreased weights (Fig. 5d, f, and h).

**Fig. 6:**
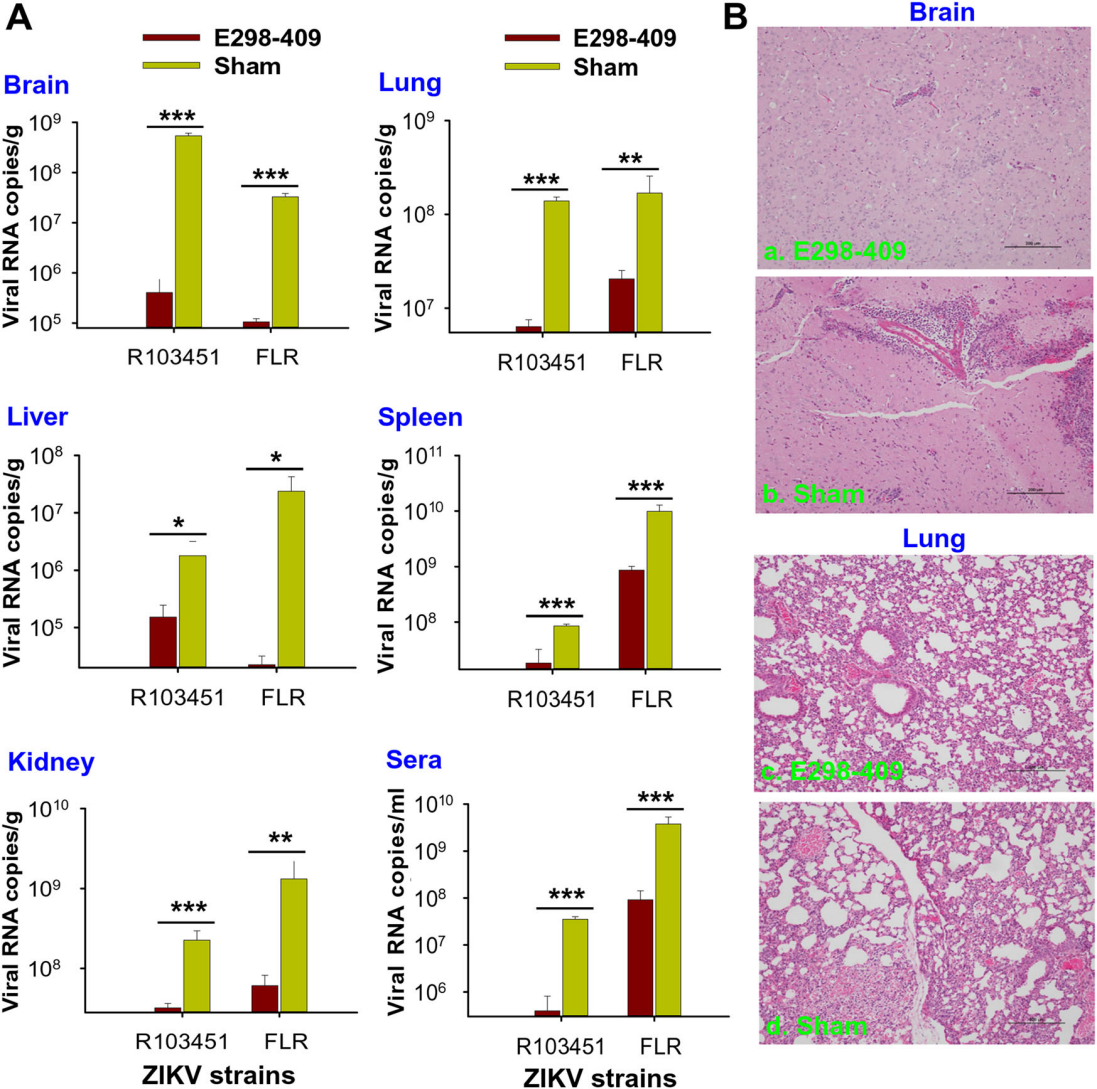
Adoptive transfer of sera of mice immunized with E298-409 of ZIKV EDIII significantly alleviated viral replication and pathological effects in ZIKV-infected A129 mice (**A**) Five-week-old A129 mice (*n* = 6) were passively transferred with anti-E298-409 sera (e.g., 10^5^ ZIKV EDIII-specific IgG antibody titer), challenged with ZIKV R103451 or FLR (10^3^ PFU) strains 6 h before and 12 h after infection, and detected for ZIKV RNA by qRT-PCR in tissues (brain, lung, kidney, liver, and spleen) and sera at 3 days p.i. The data are presented as the means ± SE of six mice in each group. The experiments were repeated twice and similar results were obtained. Significant differences (*P < 0.05; **P < 0.01; ***P < 0.001) were seen between E298-409 and sham control groups in the test tissues and sera. (**B**) Five-week-old A129 mice were passively transferred with anti-E298-409 sera (a, c), infected with ZIKV R103451 as above, and detected for pathological changes at 5 days p.i. Representative images from mouse brain (a, b) and lung (c, d) tissues are shown. The H&E-stained tissue sections were observed under light microscopy (×10 magnification). Sham control, A129 mice infected with ZIKV R103451

The ability of anti-E298-409 sera to inhibit ZIKV replication was evaluated in A129 mice by qRT-PCR at 3 days p.i. to detect viral titers in tissues and sera and by H&E staining at 5 days p.i. to evaluate pathological changes in tissues. The qRT-PCR results demonstrated that mice receiving anti-E298-409 sera had significantly reduced viral titers in tissues, including brain, lung, liver, spleen, and kidney, and sera after challenge with ZIKV R103451 or FLR strain, compared with the sham control mice that did not receive sera (Fig. 6A). Analysis of pathological changes revealed that control A129 mice had severe brain damage with enhanced neuronal eosinophilia, neuronal necrosis, T-cell proliferation, and focal bleeding in the brain tissues (Fig. 6Bb), as well as serious interstitial inflammation, focal bleeding, epithelial cell degeneration, and inflammatory cell infiltration in the lung tissues (Fig. 6Bd). In contrast, the anti-E298-409 sera-treated A129 mice demonstrated considerable alleviation of tissue damage, along with significantly decreased neuronal eosinophilia and T-cell proliferation in the brain tissues (Fig. 6Ba) and slight interstitial inflammation, epithelial cell degeneration, and inflammatory cell infiltration in the lung tissues (Fig. 6Bc).

This line of evidence confirmed that the protection of A129 mice against ZIKV challenge was positively correlated with the amount of neutralizing antibodies, suggesting that the protection is mediated by the EDIII-specific neutralizing antibodies. These results further demonstrated the protective efficacy of the identified EDIII fragment against divergent human epidemic ZIKV strains.

## Discussion

In this study, we explored the potential for developing effective and safe ZIKV EDIII-based vaccines with potent neutralizing activity and protective efficacy against ZIKV infection. By comparing the cross-neutralization and protection of three recombinant vaccines encoding ZIKV EDIII fragments of different lengths, we identified a critical neutralizing fragment containing residues 298–409 of EDIII (e.g., E298-409) that induced the highest titers of cross-neutralizing antibodies against infection with nine ZIKV strains with different or the same EDIII sequences isolated from different hosts, regions, and time periods. The identified E298-409 fragment with the highest neutralizing potential had the protective efficacy to protect newborns and lethal adult A129 mice against the two epidemic human strains of ZIKV tested. These data suggest that the N- and C-terminal residues of ZIKV EDIII have important roles in inducing protective neutralizing antibodies and that an appropriate extension of EDIII residues to the N-terminal DI region and C-terminal stalk region (Supplementary Figure S1) helps in the production of effective neutralizing antibodies against ZIKV infection.

Previous studies have identified several ZIKV EDIII-specific mouse neutralizing mAbs, such as ZV-54^36^. Here, we found that, similar to ZV-54 mAb, which maintained the highest neutralizing capacity (the lowest ND_50_) in neutralizing R103451 (Supplementary Figure S5), a recent human ZIKV strain, anti-E298-409 pAb also had the highest neutralizing activity against this viral strain (Fig. 1). In addition, both EDIII-specific pAb and mAb can effectively neutralize other epidemic human ZIKV strains tested, including FLR, PAN2015, PAN2016, and PRVABC59 (Fig. 1; Supplementary Figure S5), suggesting a broad neutralizing capacity of ZIKV EDIII-specific antibodies against infection with ZIKV, particularly the pandemic human strains. As a number of variations have been demonstrated in the different strains of ZIKV tested, including viral E proteins (Supplementary Figure S4), the ability of the identified critical neutralizing domain of EDIII (e.g., E298-409) to induce cross-neutralization and protective efficacy will be beneficial and provide useful guidance for the development of effective vaccines to protect against current and future epidemic ZIKV strains.

ZIKV infection remains a serious health threat to pregnant women due to the ability of ZIKV to cross the placenta and infect fetuses, leading to severe birth defects^3–5,39^. Therefore, it is critically important to develop vaccines with potent efficacy to protect pregnant women, as well as their fetuses and newborns, against ZIKV infection, reducing the occurrence of CZS, particularly microcephaly. In this study, we evaluated the ability of ZIKV EDIII fragments to protect against ZIKV infection in pups born to immunized female mice. We confirmed that the pups born to E298-409-immunized mice had highly potent neutralizing antibody responses and were fully protected against lethal ZIKV challenge. These results suggest that the neutralizing antibodies in fetuses transferred via placenta from immunized pregnant mothers are effective in protecting the newborns from ZIKV infection, thus providing evidence to show the effectiveness of immunizing pregnant women with an EDIII-based vaccine for the protection of fetuses or newborns against ZIKV infection.

Protective efficacy against ZIKV is mainly associated with neutralizing antibodies targeting the viral E protein^23,36,40,41^. Indeed, the present study has demonstrated that passive transfer of ZIKV EDIII-specific neutralizing antibodies could protect naive newborns against ZIKV infection. Particularly, adoptively transferred neutralizing mouse sera specific to the identified E298-409 fragment conferred to newborns complete protection against lethal challenge with two epidemic human ZIKV strains. Such sera with neutralizing activity were also protective against infection with the above two ZIKV strains in type I interferon receptor-deficient adult A129 mice, significantly preventing viral replication and increasing mouse survival. These results suggest that E298-409-specific neutralizing antibodies can effectively protect normal newborn babies and immunocompromised adults from ZIKV infection and that protection against ZIKV infection is positively associated with the amount of these neutralizing antibodies.

Overall, this study has identified a critical fragment overlapping ZIKV EDIII, E298-409, for developing effective and safe ZIKV vaccines. The expressed protein containing E298-409 shows promise for further development as an efficacious and safe vaccine for prevention of ZIKV infection in high-risk populations, particularly pregnant women and their fetuses. E298-409-specific neutralizing antibodies may also be used for pre-exposure prophylaxis of ZIKV infection in newborn babies and immunocompromised adults.

## Electronic supplementary material

Supplementary materials
