# Supplementary material for: Critical neutralizing fragment of Zika virus EDIII elicits cross-neutralization and protection against divergent Zika viruses
Source: Emerg Microbes Infect. 2018 Jan 24;7:7. doi: 10.1038/s41426-017-0007-8 (PMC5837162; doi:10.1038/s41426-017-0007-8)
Supplement: Supplementary file 1 — Supplementary materials [file 41426_2017_7_MOESM1_ESM.doc]

**Supplementary Materials**

**Supplementary Figure S1** Structures of ZIKV E protein and construction of ZIKV EDIII protein fragments. (**A**) Schematic structure of ZIKV E protein. DI, DII, and DIII, domain I, II, and III of E protein. FL, fusion loop. S, stalk region. TM, transmembrane domain. (**B**) Constructed EDIII protein fragments containing residues 296-406, 298-409, and 301-404 of E protein, respectively, with a C-terminal Fc of human IgG1. (**C**) Structure of ZIKV E protein (PDB 5IRE). (**a**) Monomeric subunit of ZIKV E protein. DI is in red, DII in yellow, DIII in blue, stalk in magenta, and transmembrane anchor in pink. (**b**) EDIII constructs. The domain boundaries of the different constructs are labeled. A small portion of the beta-strand from DI is shown in red, and a small portion of the alpha-helix from the stalk is shown in magenta.

**
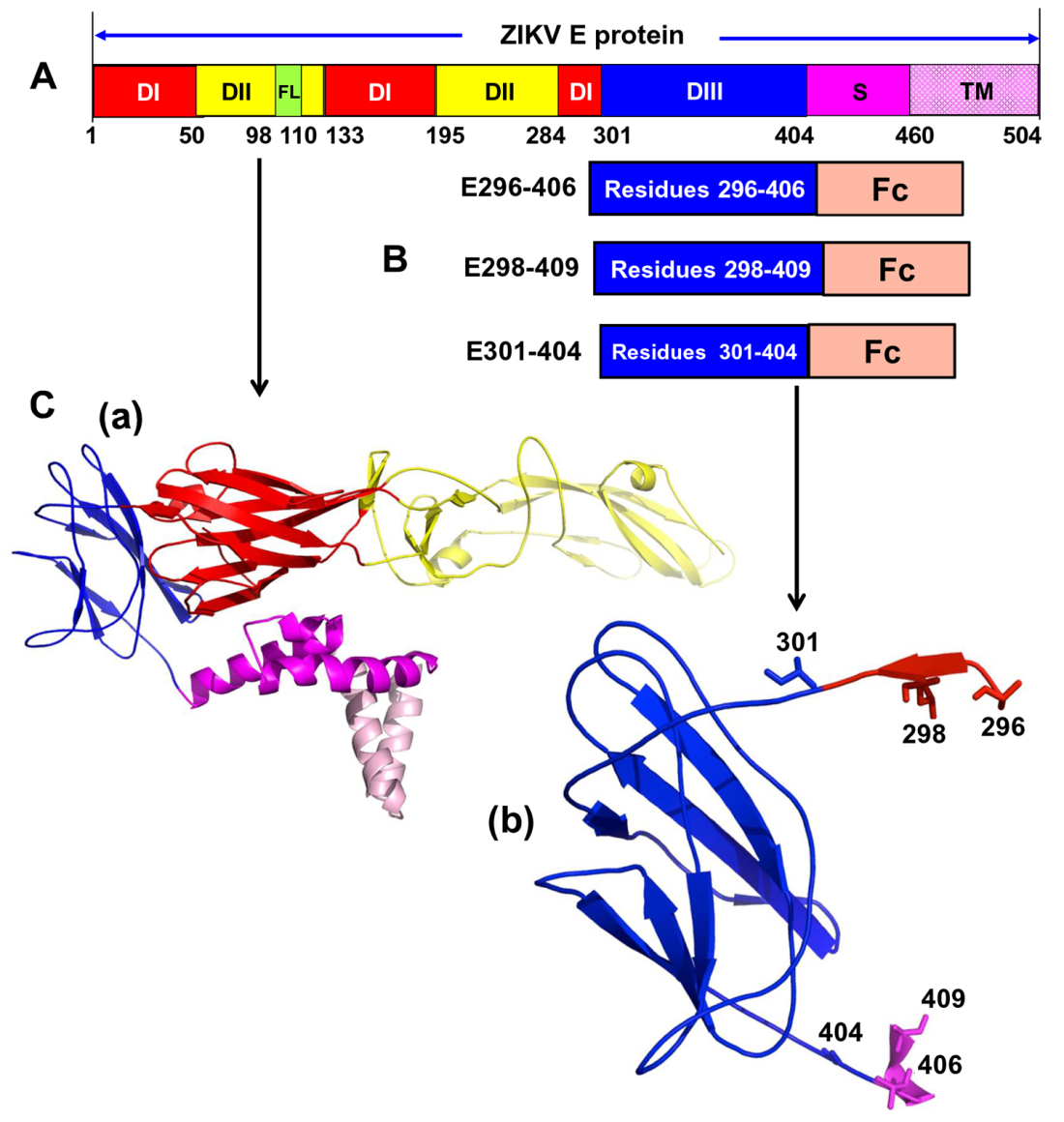
**

**Supplementary Figure S2**  Characterization of ZIKV EDIII protein fragments. SDS-PAGE (**A**) and Western blot (**B**) analysis of expressed proteins. Samples (5 μg), either boiled or non-boiled, were subjected to SDS-PAGE, followed by Coomassie Brilliant blue staining, or Western blot using ZIKV EDIII-specific mAb, ZV-54 (0.4 μg/ml). The protein molecular weight marker (kDa) is indicated on the left.

**
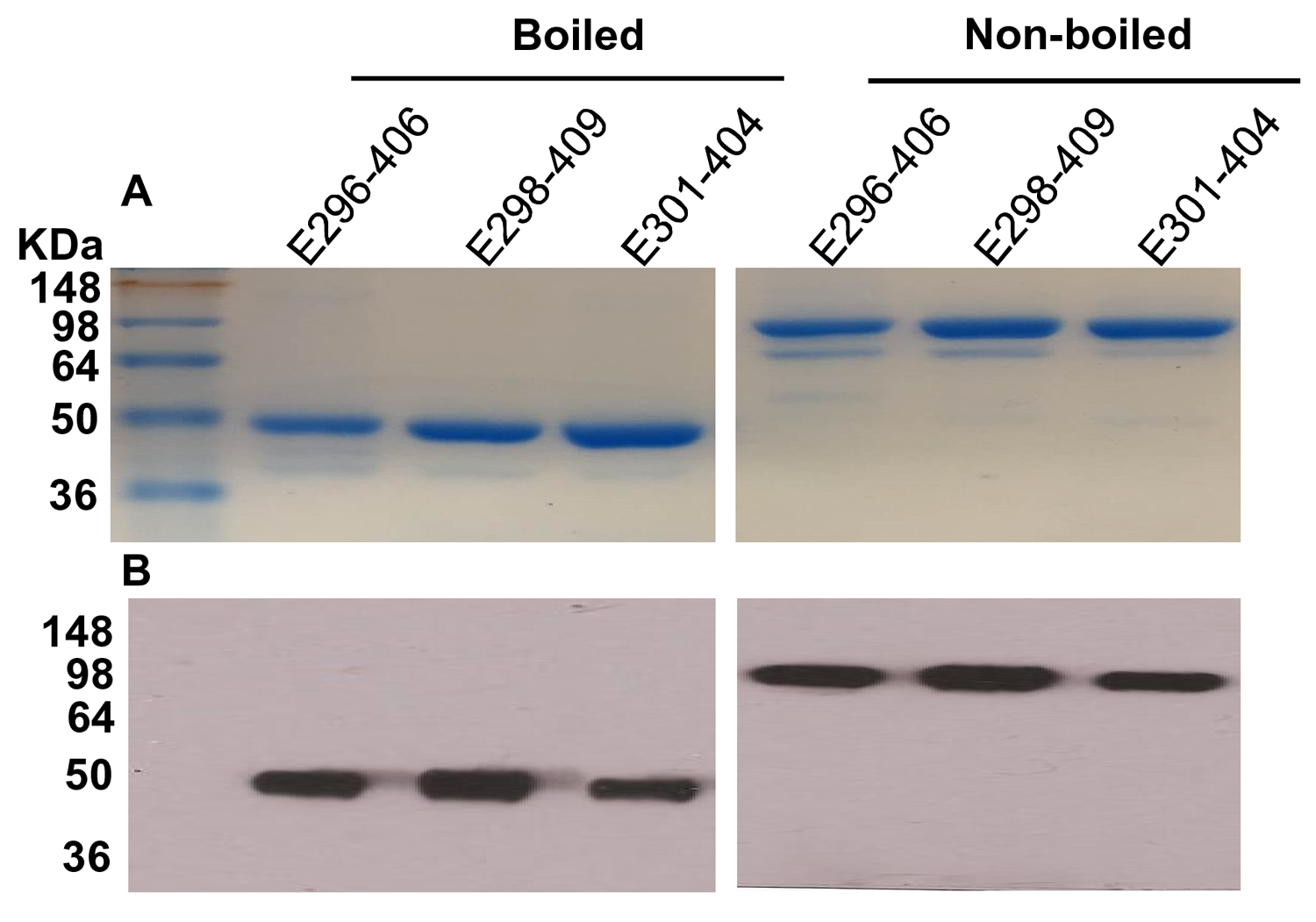
**

**Supplementary Figure S3** Immunization and challenge schedules of ZIKV EDIII protein fragments. (**A**) Immunization schedule. Female BALB/c mice were respectively i.m. immunized with EDIII protein fragments, or PBS control, plus Alum and MPLA adjuvants, as described in Materials and Methods. Mice were boosted with the same immunogens at day 21, day 42, month 7, and month 10, and sera were collected at month 0, 2, 7 and 10 post-immunization to detect ZIKV EDIII and E-specific antibody and anti-ZIKV neutralizing antibody. (**B**) Challenge schedule. At month 7, immunized female mice were mated with naïve male BALB/c mice, and the generated 7-day-old pups were challenged with ZIKV. Sera collected at month 7 or month 10 were passively transferred into 7-day-old pups born to naïve BALB/c mice and 5-week-old A129 mice, respectively, and challenged with ZIKV, as described in Materials and Methods.

**
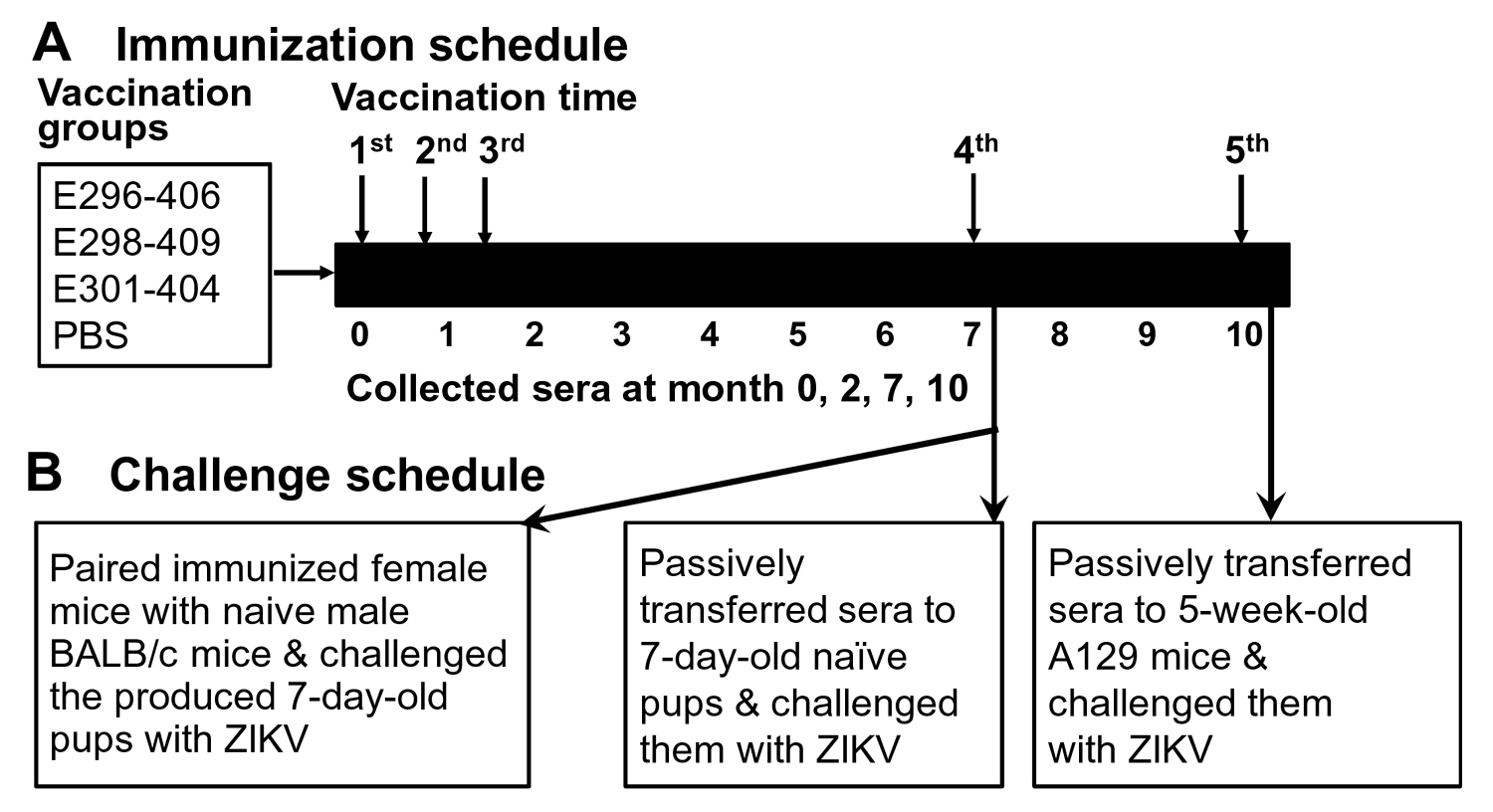
**

**Supplementary Figure S4** Comparison and multiple sequence alignment of amino acid sequences of ZIKV strains used for the studies. (**A**) Information of ZIKV strains used for the neutralization test. (**B**) Phylogenetic tree of the ZIKV strains listed. The tree was built by using the neighbor-**joining method** with 1,000 bootstrap values calculated from 100 trees in MEGA (v6.0). The tree was midpoint rooted. The scale bar indicates the estimated numbers of substitutions per 100 nucleotides. (**C**) Multiple sequence alignment of amino acid sequences of E proteins of the ZIKV strains listed using Clustal X software (v2.0).

**
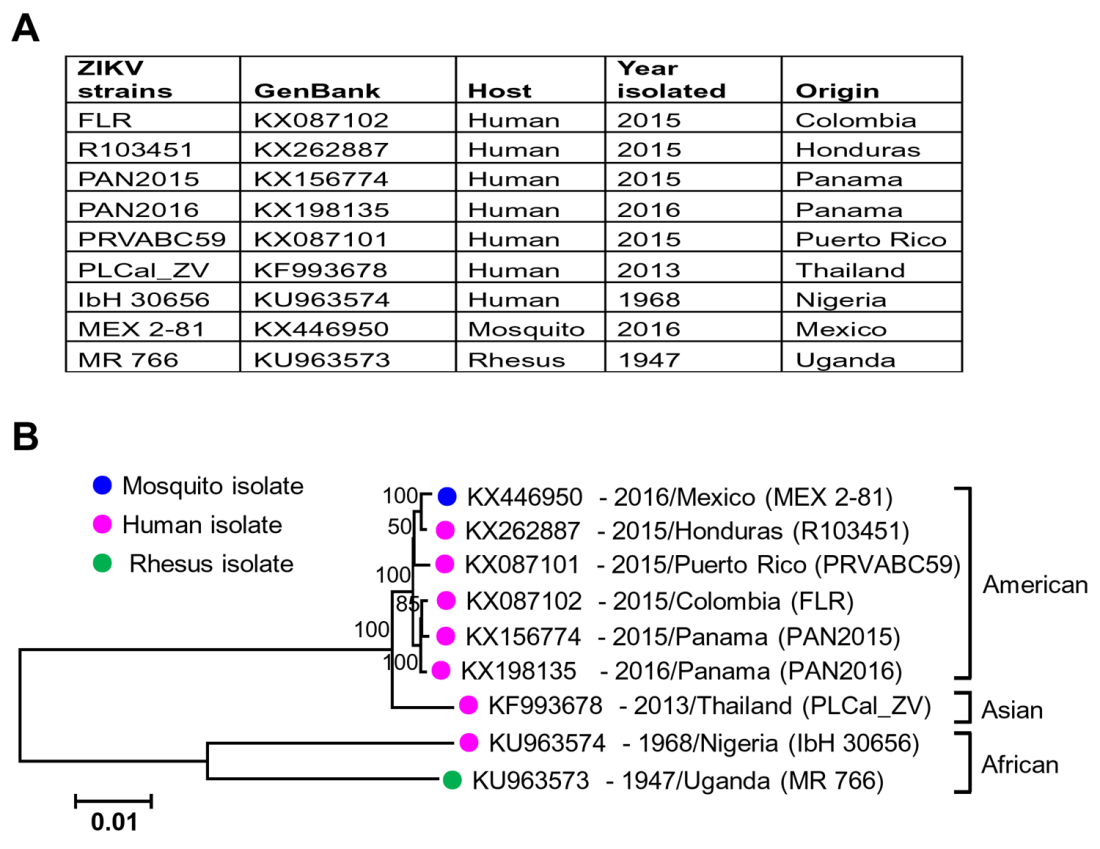
**

**C**

**
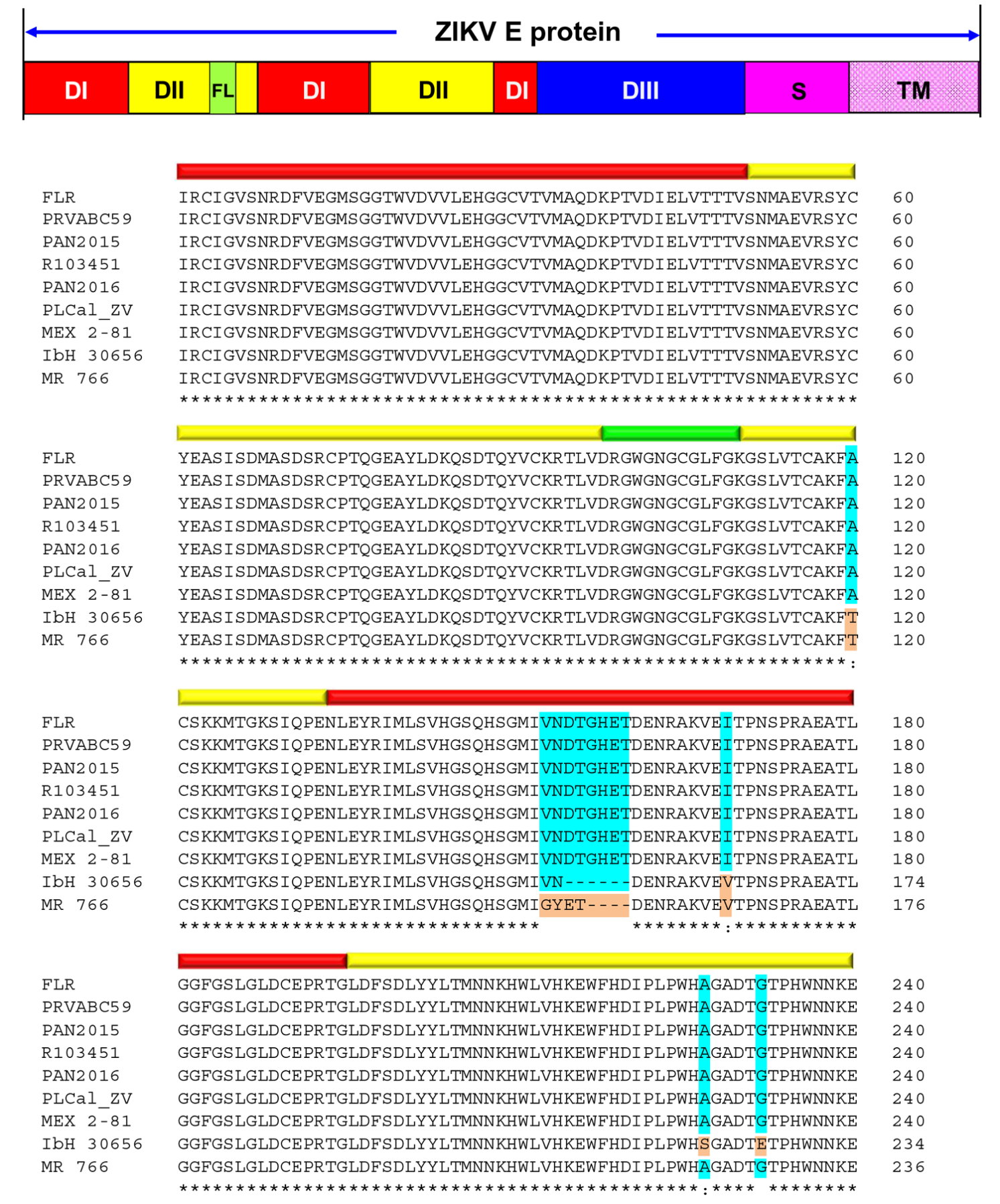
**

**
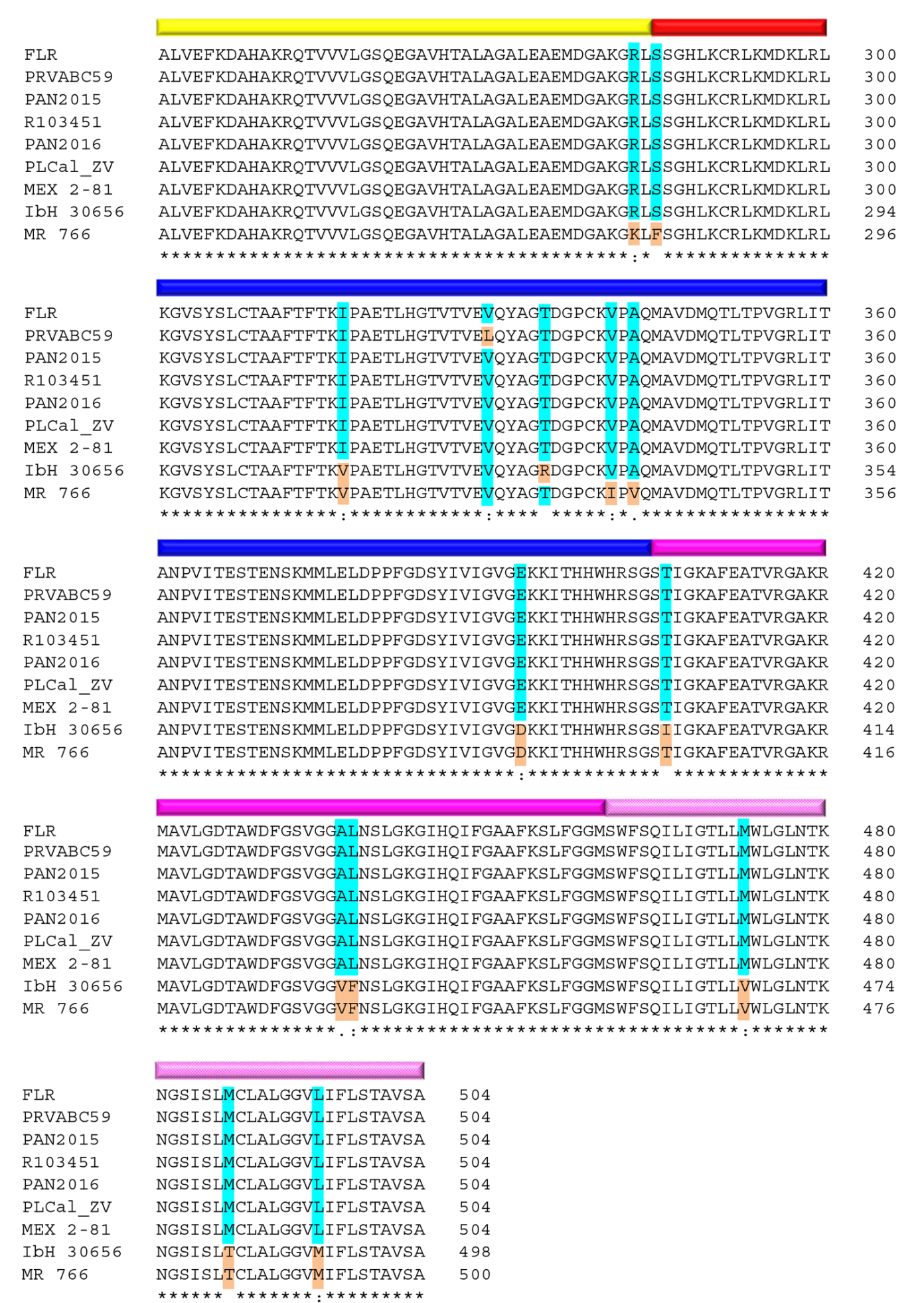
**

**Supplementary Figure S5** Neutralizing activity of ZV-54 mAb against ZIKV. The mAb was tested by PRNT assay against the aforementioned divergent ZIKV strains isolated from human, mosquito, and rhesus. The neutralizing activity of mAb was calculated in duplicate wells at 5-fold serial dilutions from 50 μg/ml, and the data are presented as mean 50% neutralization dose (ND_50_) ± SE of duplicate wells.

**
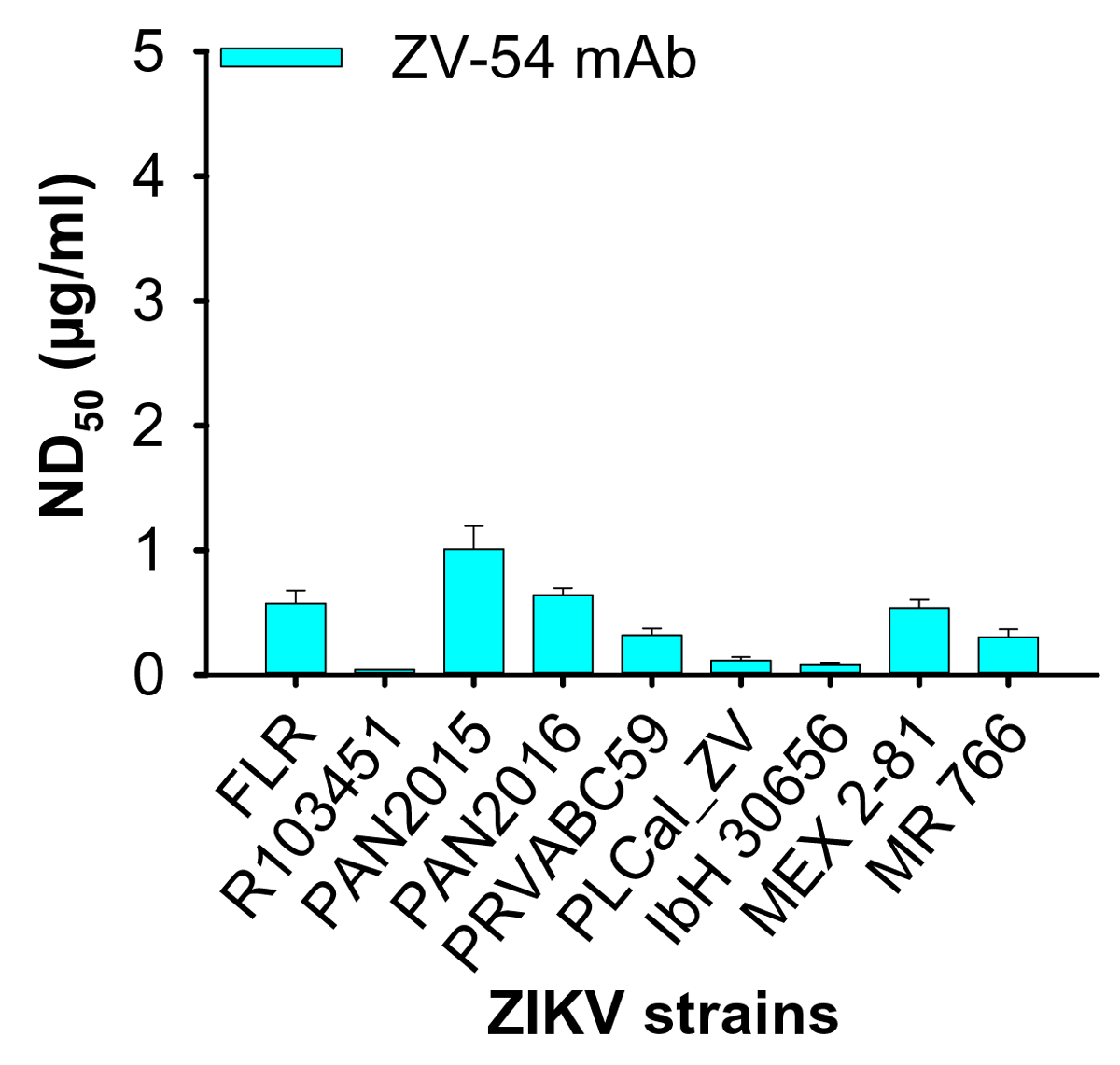
**
